# Impact of Pesticide Exposure on High-Frequency Auditory Thresholds and Cochlear Function in Young People Residing near Agricultural Areas

**DOI:** 10.3390/toxics13050375

**Published:** 2025-05-06

**Authors:** Felipe Munoz, Cristian Aedo-Sanchez, Felipe Paredes-Aravena, Enzo Aguilar-Vidal, Pedro Jilberto-Vergara, Gonzalo Terreros

**Affiliations:** 1 Laboratorio de Neurociencia Sensorial Perceptual y Cognitiva, Instituto de Ciencias de la Salud, Universidad de O’Higgins, Rancagua 2820000, Chile; felipe.munoza@uoh.cl (F.M.); pedrojilberto.v@gmail.com (P.J.-V.); 2 Programa de Doctorado en Ciencias e Ingeniería para la Salud, Universidad de Valparaíso, Valparaíso 2520000, Chile; 3 Departamento de Tecnología Médica, Facultad de Medicina, Universidad de Chile, Santiago 8380453, Chile; caedo@uchile.cl (C.A.-S.); eaguilar@uchile.cl (E.A.-V.); 4 Escuela de Tecnología Médica, Facultad de Medicina, Universidad de Chile, Santiago 8380453, Chile; 5 Escuela de Salud, Universidad de O’Higgins, Rancagua 2820000, Chile; felipe.paredesa@pregrado.uoh.cl

**Keywords:** pesticides, hearing loss, ototoxicity, distortion product otoacoustic emissions, auditory brainstem response

## Abstract

Pesticide exposure poses a significant health risk, with emerging evidence suggesting its impact on auditory function. This study investigated the effects of pesticide exposure on hearing in young rural residents living near agricultural areas in Chile’s O’Higgins Region. We evaluated 51 participants (31 exposed, 20 unexposed) aged 18–35 years, using comprehensive audiological assessments including high-frequency audiometry, distortion product otoacoustic emissions (DPOAEs), and auditory brainstem responses (ABR). Participants were classified based on residential proximity to agricultural areas, with exposed individuals living around 400 m or less from monoculture fields. Results revealed significant differences in high-frequency hearing thresholds (14 and 16 kHz) in exposed individuals. The exposed group showed a higher number of absent DPOAEs and reduced ABR wave V amplitude in the right ear compared to the unexposed group. Additionally, the wave V/I ratio was significantly lower in exposed individuals. These findings suggest that pesticide exposure, even without direct occupational contact, may induce measurable changes in auditory function, particularly affecting high-frequency hearing and neural responses. These results emphasize the need for enhanced monitoring and protective measures for populations residing near agricultural areas where pesticides are extensively used.

## 1. Introduction

Hearing impairment, a prevalent disability in contemporary society, constitutes a substantial global health challenge. It has been proposed that by 2050, one in ten people worldwide will experience disabling hearing loss [1]. Beyond its evident impact on communication abilities, this condition exacts a considerable economic toll, estimated at approximately USD 750 billion annually [2]. It is crucial to investigate the etiological factors of deafness. It has been estimated that approximately half of all hearing loss is preventable [1]. Global trends in hearing loss are influenced by a variety of factors, including noise and air pollution [3], exposure to heavy metals [4], and the increasing use of agrochemicals, particularly pesticides [5].

Acute and chronic exposure to pesticides is associated with an array of health issues, from nausea and dizziness to chronic neurological problems and cancer [6,7,8,9,10]. However, pesticide exposition has been linked to sensory impacts, particularly on hearing. Studies reported by Petty et al. [11] showed damage to the eighth cranial nerve, with symptoms including nausea and tinnitus after pesticide exposition. Later research, like that by Crawford et al. [12] and Rabinowitz et al. [13], showed that people who were exposed to organophosphate (OP) pesticides had a higher chance of losing their hearing. Also, Dundar et al. [14] found cases of people suddenly losing hearing after breathing in OPs. Moreover, Guida et al. [15] observed significant hearing loss in individuals exposed to a combination of pesticides and noise, emphasizing the compounded impact of these environmental factors. This study corroborates earlier research by Teixeira and Brandao [16], documenting sensorineural hearing loss in agricultural workers exposed to pesticides. Perry and May [17] further confirmed that these effects were independent of noise exposure from agricultural machinery. On the other hand, carbaryl, a commonly used acetylcholinesterase inhibitor in agriculture, is another substance associated with auditory system damage. Prakash Krishnan Muthaiah et al. [18] identified a significant correlation between exposure to carbaryl and damage to the basal region of the cochlea in rats on postnatal day three. This finding establishes a potential link between cochlear damage and various auditory disorders observed in individuals exposed to pesticides, including central auditory processing. A pivotal challenge in diagnosing these issues stems from the complexity of detecting cochlear damage through standard clinical tests. Consequently, individuals exposed to pesticides may experience undiagnosed auditory processing problems, potentially resulting in significant, albeit subclinical, hearing loss.

Animal model studies have elucidated the impact of noise exposure on auditory capacity, revealing temporary changes without immediate or chronic loss of hair cells. Significant damage to the connections between inner hair cells (IHCs) and cochlear neurons has been observed [19,20,21]. Notably, this reduction in neural connections may not be apparent in conventional hearing tests, which typically measure the perception of weak sounds or utilize otoacoustic emissions testing. Conversely, noise-induced hearing loss primarily affects the outer hair cells (OHCs) before impacting other cochlear areas [22], with animal studies revealing varying degrees of OHC changes after exposure to OP, ranging from mild alterations to severe damage or loss [7]. These changes are associated with reduced auditory sensitivity and frequency discrimination ability [23]. Consequently, standard hearing assessments may lack the sensitivity to detect this type of synaptic loss. Even a small number of intact IHCs can facilitate tone detection in quiet environments [19,24]. This reduction in synaptic connections, termed cochlear synaptopathy, has been linked to various factors, including noise exposure, aging, and ototoxic substances [21,25,26].

We presume that a similar process can occur with exposure to pesticides in inhabitants that live near agricultural areas, potentially inducing subtle alterations in auditory processing initially undetectable through standard hearing tests. Our primary hypothesis is that environmental pesticide exposure through residential proximity to agricultural areas can induce auditory changes that might not be detected by conventional audiometric assessments. The O’Higgins Region stands out for its significant importance in the Chilean agricultural sector. Its contribution to the national agricultural and forestry sector represents 18.6% of this sector’s Gross Domestic Product (GDP), while at the regional level, agricultural and forestry activities constitute 12.3% of the regional GDP [27]. The region is particularly notable in monocultures fields, particularly related to fruit production, concentrating 24.8% of the national area dedicated to these crops. In this region, agricultural land use reveals a specific composition of crop types. Fruit orchards occupy 21.5% of the total agricultural area, representing the largest crop category. Cereals cover 15.6% of the agricultural land, while vineyards and vine plantations account for 9.9%. Collectively, these three crop types constitute 47% of the region’s total agricultural surface, demonstrating the prominence of fruit production, cereal cultivation, and viticulture in the region’s agricultural sector [27]. The region has 8.2% of the country’s land area dedicated to cultivation, and this intense agricultural activity and high concentration of monoculture fields characterize the region as an area with significant exposure to intensive agricultural practices [27]. Specifically, in 2019, this region accounted for over half (51.23%) of all declared pesticide sales nationwide, with a volume of 28,019,055.99 kg/L out of the country’s total sales of 54,451,488.15 kg/L [28]. This high volume of pesticide sales is notable because the region exhibits a striking disparity between its agricultural area and pesticide application. Analysis of 2019 pesticide sales data from Chile’s O’Higgins Region [28] reveals that sulfur dominates the market. Among the pesticides described as harmful to the auditory system [5,29,30,31], chlorpyrifos is the most used organophosphate, and lambda-cyhalothrin is the predominant pyrethroid. Although the O’Higgins Region occupies only 8.2% of the national land dedicated to crops, it accounts for approximately 51.23% of the total pesticide use in the country [28,29], demonstrating a remarkably high intensity of agrochemical application.

This study investigates the effects of pesticide exposure associated with living near monoculture fields on auditory function in a cohort of young rural residents, evaluating audiometric thresholds, suprathreshold auditory brainstem responses (ABRs), and distortion product otoacoustic emissions (DPOAEs). By examining these measures, this research aims to elucidate the nuanced impact of pesticide exposure on auditory health, highlighting the need for further investigation to fully comprehend the complex relationship between these exposures and auditory dysfunction and ultimately to inform preventative strategies for vulnerable populations.

## 2. Materials and Methods

All evaluations were conducted in a sound-attenuated chamber with an average ambient noise level of 30 dB. An expert audiologist performed them. Neither the participants nor the experimenter knew to which group the participants belonged. While participants were informed about the general focus of the study during the consent process, they were not aware of the specific distance criteria that would determine their classification as exposed or unexposed. The group assignment was conducted only after completing all audiological evaluations and was based on GIS analysis of residential proximity to agricultural areas, information that was not revealed to participants during the assessment phase.

### 2.1. Subjects

Sixty-three Chilean volunteers, aged 18 to 35 years and native Spanish speakers, participated in this study. Participants were recruited through online platforms with messaging focused on a general environmental health study examining various factors affecting residents in the O’Higgins Region. The specific focus on pesticide exposure was not emphasized in recruitment materials to avoid introducing bias or allowing participants to self-classify their exposure status. Upon arrival, all participants completed our comprehensive questionnaire (Supplementary Material S1). After analysis of these questionnaires, 12 individuals were excluded, as they did not meet our pre-established inclusion criteria. This resulted in a final sample of 51 participants. Following data collection, participants were categorized into exposed (E) and unexposed (UE) groups based on their residential proximity to agricultural areas. Evaluations were conducted blindly, with group assignments occurring after completing all assessments. This specific age range (18–35 years) was selected to minimize the potential confounding effects of age-related hearing loss and more accurately assess pesticide exposure’s impact on auditory function. Ethical approval for this study (project number 221-2020) was granted by the Ethical and Scientific Committee for Research on Human Subjects of the University of Chile, adhering to the principles outlined in the Declaration of Helsinki. All participants provided written informed consent.

We applied the following inclusion criteria to determine participant eligibility for the experimental group: people who lived within a radius approximately 400 m or less from the monoculture areas in the last 3 years and people who were 35 years old or younger. On the other hand, individuals with a medical history of persistent otitis during childhood, adolescence, and adulthood; those whose workplaces exposed them to constant or variable noise levels equal to or exceeding 80 dB; candidates who shared their homes with family members who worked in monoculture fields; volunteers involved in the management and cultivation of residential gardens and using agricultural chemicals; and people who had worked at least 1 year in agricultural activities were all excluded. Additionally, individuals with neuropsychiatric conditions that could lead to heightened sensitivity to auditory stimuli and who chronically consumed ototoxic medications or substances linked to hearing loss were also ineligible [32]. Finally, people who had had accidents that caused traumatic tympanic membrane ruptures or who had visible ruptures and/or a Type B tympanogram, earwax blockage, or a Pure-Tone Average (PTA), calculated as the average of hearing thresholds at 0.5, 1, and 2 kHz, with a value of 20 dB or higher were omitted. We applied this PTA threshold exclusion criterion to establish a baseline of normal hearing in conventional audiometric frequencies across all participants. This approach allowed us to control pre-existing hearing loss from various potential causes unrelated to our research question. By ensuring all participants had clinically normal hearing in these conventional frequencies, we created a methodologically sound foundation to investigate the more subtle effects of pesticide exposure across the entire frequency spectrum, including high frequencies that are not part of standard clinical assessments. This design choice was based on previous research suggesting that toxic exposures often first manifest in high-frequency regions before affecting conventional frequencies [7,19,24,33]. This exclusion criterion helped establish comparable groups at baseline regarding conventional hearing thresholds, allowing us to better isolate potential effects of pesticide exposure. While we acknowledge this approach may have excluded some individuals with pre-existing hearing loss possibly related to pesticide exposure, it reduced potential confounding from other causes of hearing impairment, enabling a more focused analysis of the relationship between pesticide exposure and auditory function in participants with normal clinical hearing.

**Figure 1:**
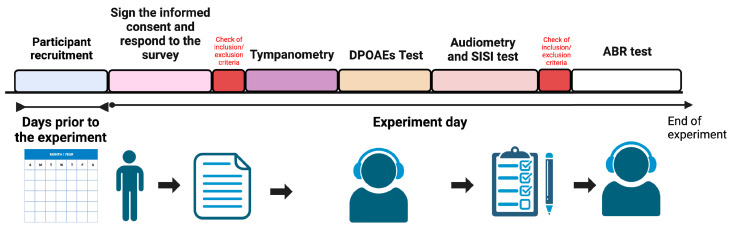
Timeline of Experimental Milestones. This figure graphically represents the key chronological events of the experiment. The process commenced with the recruitment of participants. Upon arrival of the participants, informed consent was obtained, after which data were collected by means of a survey. Subsequently, inclusion and exclusion criteria related to prior illnesses or disqualifying conditions were assessed. The tympanometry followed this, along with distortion product otoacoustic emissions (DPOAEs) testing, audiometry, and the short increment sensitivity index (SISI) test. Criteria for inclusion and exclusion of auditory conditions were then re-evaluated. Finally, auditory brainstem response (ABR) tests were performed. Created in BioRender. Terreros, G. (2025) https://BioRender.com/h47u910 (accessed on 8 February 2025).

For the control (UE) group, we applied the same inclusion and exclusion criteria as for the E group, with two key differences: participants in the control group lived more than 400 m away from monoculture areas in the last 3 years. All other criteria, including age restrictions, medical history requirements, and audiological conditions, remained the same for both groups. This ensured that the primary difference between the groups was their pesticide exposure through proximity to agricultural areas. The experimental protocol is shown in Figure 1.

### 2.2. Experimental Groups and Pesticide Exposition

Upon arrival, participants completed a comprehensive questionnaire that collected information on general health status, auditory health history, socioeconomic background, biographical data, occupational history, and detailed pesticide exposure, including household insecticide use and personal gardening practices. This questionnaire, presented in translated form in Supplementary Material S1, underwent pilot testing to ensure clarity, validity, and reliability. The survey assessed previous exposure to ototoxic substances, history of acoustic trauma, occupational noise exposure, family history of hearing disorders, previous ear pathologies, and current auditory symptoms. This information, combined with our exclusion criteria, enabled us to control factors that might affect auditory function independently of pesticide exposure.

**Figure 2:**
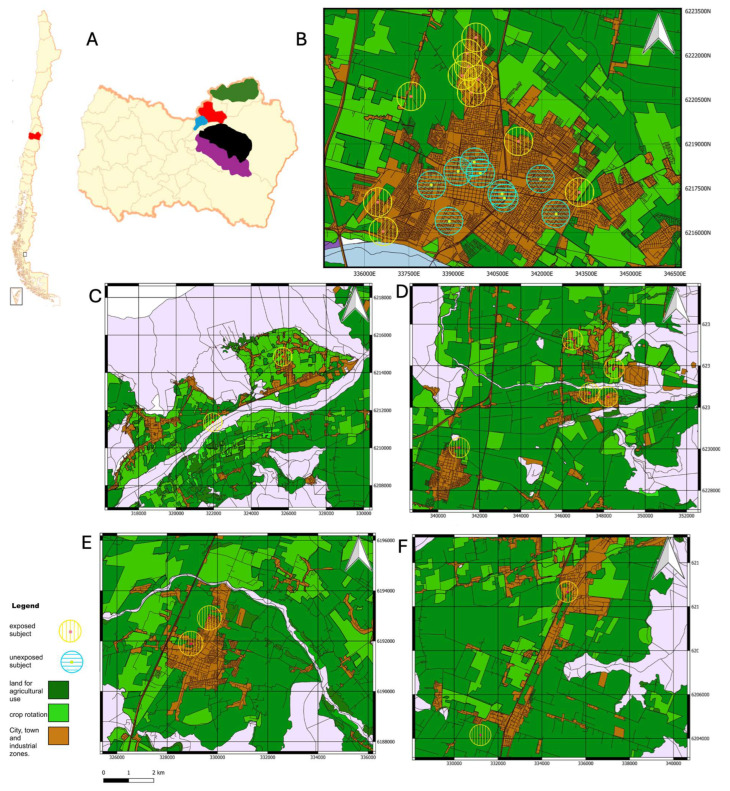
Geographic Representative Distribution of Participants Across Various Localities in the O’Higgins Region, Chile. This composite figure consists of six panels (**A**–**F**). Panel (**A**) shows two maps: a small map of Chile (**left**) with the O’Higgins Region highlighted in red, and a larger map of the O’Higgins Region (**right**) with color-coded areas indicating the localities detailed in subsequent panels: Rancagua (red), Doñihue (blue), Mostazal (green), Rengo (purple), and Requínoa (black). Panels (**B**–**F**) show detailed maps of each locality. The legend indicates exposed subjects (yellow circles with vertical lines), unexposed subjects (blue circles with horizontal lines), monoculture fields (represented in two types: permanent agricultural land (dark green) and seasonal crop rotation areas (light green)), and city/town/industrial zones (brown). White areas represent water bodies, and grey areas represent non-agricultural land. (**B**) Geographic distribution of participants in Rancagua, showing the urban center (brown) surrounded by monoculture fields with both exposed and unexposed participants. (**C**) Participant distribution in Doñihue. (**D**) Participant distribution in Mostazal. (**E**) Participant distribution in Rengo. (**F**) Participant distribution in Requínoa. Note that while the main maps (Panels (**B**–**F**)) are presented in a rectangular format for cartographic clarity, the corresponding highlighted areas on the regional overview map (Panel (**A**)) represent the actual municipal boundaries of each locality, which are irregularly shaped. The areas of each municipality highlighted on the regional map are larger and show the total size of each commune. The maps in Panels (**B**–**F**) are rectangular and show a smaller portion of territory because they are focused on representing the distance of participants from those communes to monoculture fields.

To categorize participants based on their residential history over the past 3 years, we used Geographic Information System (GIS) software (QGIS version 3.30.0) to calculate the distance between their residences and nearby monoculture fields. This analysis, visualized in a geographic map depicting agricultural monoculture fields and urban settlements (Figure 2), utilized two layers: the 2017 Chilean Census (the latest available at the time of publication) and the “Land Use Change Monitoring in the O’Higgins Metropolitan Region, 2016” layer from the IDE [26] to establish monoculture locations and participant addresses. Following the proximity criterion established by Fenske et al., [28] participants residing within approximately a 400-m radius of a monoculture field were classified as exposed (E). This threshold was selected due to the prevalence of organophosphate (OP) pesticide spraying in the region. A geo-referenced map (Figure 2) illustrates participant residences in relation to the nearest agricultural fields. Several cities in the region, including Rancagua, Doñihue, Mostazal, Rengo, and Requinoa, are characterized by residential areas located close to agricultural zones, with many neighborhoods falling within the exposure radius of monoculture fields. This classification, based on residential proximity to monoculture fields, is crucial for understanding the potential impact of agricultural spraying and related activities. It strengthens the study’s ability to attribute observed differences in auditory measures between groups more confidently to pesticide exposure, rather than to general rural–urban lifestyle differences or other environmental factors, thereby playing a key role in interpreting subsequent findings related to auditory health and audiological risks associated with agricultural environments.

The classification allowed us to have two groups: the “E” group (n = 31) with a mean age of 25.94 ± 6.45 years and the “UE” group (n = 20) with a mean age of 28.2 ± 5.34 years, showing no significant age difference (Mann–Whitney test; *p* = 0.152). The sex distribution showed no significant differences (Fisher’s exact test; *p* = 0.147), with the E group consisting of 10 males (32.26%) and 21 females (67.74%), while the UE group had 11 males (55%) and nine females (45%). No significant differences were found in economic perception between groups (chi-square test; *p* = 0.502) with respect to socioeconomic factors. In the E group, 13 participants (41.94%) reported high economic status, 16 (51.61%) medium, and 2 (6.45%) low, while in the UE group, 11 (55%) reported high status, 7 (35%) medium, and 2 (10%) low. Lifestyle factors were also comparable between groups, showing no significant differences in alcohol consumption (Fisher’s exact test; E: 54.84% yes, 45.16% no; UE: 60% yes, 40% no; *p* = 0.778) or smoking habits (E: 22.58% yes, 77.42% no; UE: 30% yes, 70% no; *p* = 0.743). Table 1 shows the population details of the subjects participating in the study.

We specifically excluded individuals with workplace noise exposure ≥ 80 dB, chronic exposure to ototoxic medications, history of recurrent ear infections, recent acoustic trauma, family history of early onset hearing loss, or neurological conditions affecting auditory processing.

### 2.3. Hearing Assessment

#### 2.3.1. Audiogram Thresholds

We captured air thresholds between 125 and 16,000 Hz using an AC40 audiometer (Interacoustics™^®^, Middelfart, Denmark) with DD45 headphones. Our approach adhered to the clinical standards outlined by ANSI S3.6, 2010. To check the exclusion criteria, we calculated the PTA hearing threshold for frequencies 0.5, 1, and 2 kHz. Then, we determined the audiometric threshold to 16 KHz. The Short Increment Sensitivity Index (SISI) test was used to evaluate the ability to detect intensity increments. For the SISI test, we aimed to identify cochlear injury with a specific frequency 20 dB above the audiometric threshold for 2 min. A total of 20 distinct sound modulations occurs within this two-minute testing window. The SISI assessment is iterated across frequencies of 500, 1000, 2000, 4000, and 8000 Hz. Participants are directed to signal any perceived variations in loudness through a response trigger linked to a counter.

#### 2.3.2. Distortion Product Otoacoustic Emissions (DPOAEs)

To quantify DPOAEs, we employed the Titan Interacoustics system. Primary tones (f1 and f2) were introduced at 65 and 55 dB SPL, respectively, with an f2/f1 ratio of 1.22. We employed six distinct f2 frequencies: 1000, 1500, 2000, 3000, 4000, and 6000 Hz, measured in both right and left ears. The presence of DPOAEs was automatically determined by the equipment and visually confirmed by an expert audiologist.

#### 2.3.3. Auditory Brainstem Responses

Our suprathreshold ABR waves I and V measurements utilized the Eclipse EP25 instrument alongside authorized research equipment (Interacoustics™, Middelfart, Denmark) to induce the ABR response. Stimuli were presented as CE-chirps through ER-2B insert earphones at intensities of 80 dB, each lasting for 100 μs. Responses were recorded using active electrodes positioned on both mastoids and the vertex as reference points and a ground electrode placed on the lower forehead. Waves I and V were identified using two sets of 4000 repetitions and a 150/3000 Hz band-pass filter, and the recordings used had a reproducibility of 99%. To facilitate subsequent analysis, latencies and amplitudes of each participant’s waves I and V were measured. The peak-to-valley amplitudes were determined by assessing the distance between the positive and negative peaks through expert visual inspection by three independent experienced audiologists. The wave V/I ratio was calculated by dividing the amplitude of wave V by that of wave I [34]. When a wave was absent or could not be identified, it was excluded from the respective analysis.

#### 2.3.4. Statistical Analysis

Data were analyzed using Prism 8 software (GraphPad Software Inc., La Jolla, CA, USA). Normality of all variables was assessed using the Shapiro–Wilk test. Homoscedasticity was evaluated using Levene’s test. Parametric or non-parametric statistical methods were employed based on these assessments. Categorical variables, including sex distribution, work, and lifestyle factors, were analyzed using Fisher’s exact test. Economic perception was analyzed using a chi-square test. Age differences between groups were compared using the Mann–Whitney U test. On the other hand, audiometric evaluation data were analyzed using a two-way repeated measures ANOVA with group (Exposed, Unexposed) and frequency (0.15 to 16 kHz) as factors. For audiometric data, a total of 15 multiple comparisons were performed (one for each frequency), and post hoc comparisons were conducted using the Bonferroni correction. All *p*-values presented for these comparisons in the audiometric results are adjusted *p*-values as reported directly by the Prism 8 software. SISI test results were analyzed using ANOVA. Distortion product otoacoustic emissions (DPOAEs) data exhibited non-Gaussian distributions. Therefore, differences between groups were assessed using the Mann–Whitney U test (two-tailed), followed by Bonferroni post hoc correction. Latency and peak amplitude values in the ABR test were compared between groups using either Student’s *t*-test (for normally distributed data) or the Mann–Whitney U test (for non-normally distributed data), as appropriate. All results are presented as mean ± standard error of the mean (SEM) unless otherwise stated. A *p*-value of less than 0.05 was considered statistically significant.

## 3. Results

A total of 51 final participants were included in this research. All subjects were evaluated with electrophysiological and perceptual auditory tests, including tonal audiometry, high-frequency audiometry, the Short Increment Sensitivity Index (SISI) test, DPOAEs, and ABR. A rigorous health examination confirmed the absence of outer and middle ear pathologies, absence of diagnosed neurological diseases, absence of chronic pharmacological and/or ototoxic therapy, absence of exposure to intense and/or occupational noise, and absence of tinnitus.

### 3.1. Conventional Audiometry, High-Frequency Hearing Thresholds, and SISI Test

**Figure 3:**
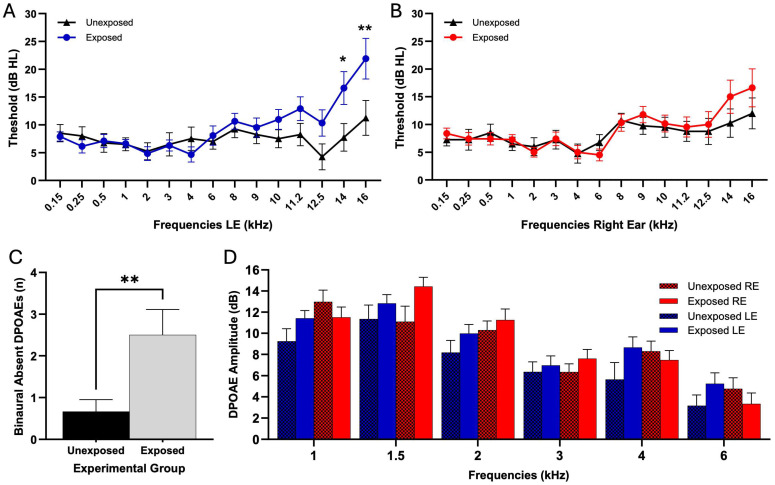
Audiometry and Distortion Product Otoacoustic Emissions (DPOAEs) in Pesticide E vs. UE Individuals. (**A**) Left ear audiometry. Individuals of the E group are represented in blue, while UE individuals are shown in black. Audiometric measurements were conducted at frequencies from 0.15 to 16 kHz. Statistically significant differences were observed at 14 and 16 kHz frequencies, where pesticide E individuals exhibited higher auditory thresholds than UE individuals (*p* < 0.05, indicated by asterisks; * *p* < 0.05, ** *p* < 0.01. Statistical significance). (**B**) Right ear audiometry: Low- and high-frequency audiometric evaluations of the right ear revealed no statistically significant differences at any frequency. (**C**) Binaural number of absent DPOAEs. Bars show the number of absent DPOAEs in both ears for frequencies between 1 and 6 kHz. A significantly higher percentage of absent DPOAEs was noted in E subjects (*p* < 0.05, indicated by asterisks; * *p* < 0.05, ** *p* < 0.01. Statistical significance). (**D**) Distortion product otoacoustic emission (DPOAE) amplitudes. This figure presents the DPOAE amplitudes for both experimental groups, displaying individual ear results across all tested frequencies. Right ear data are represented in shades of red, and left ear data are represented in shades of blue, as indicated in the figure legend. Error bars represent SEM. Asterisks (*) denote statistically significant differences (*p* < 0.05) between E and UE groups; * *p* < 0.05, ** *p* < 0.01. Statistical significance.. Abbreviations: DPOAE = distortion product otoacoustic emission; kHz = kilohertz; RE = right ear; LE = left ear.

Left ear audiometric evaluation was performed examining 15 frequencies from 0.15 to 16 kHz (Figure 3A). The results revealed significant interaction effects of pesticide exposure on frequency responses (two-way ANOVA; F (14, 733) = 1.973, *p* = 0.02). Bonferroni’s multiple comparisons test showed significant differences at 14 kHz, where the E group showed significantly higher auditory thresholds than the UE group (E: 16.61 ± 16.45 dB; UE: 7.75 ± 11.06 dB; *p* = 0.02). At 16 kHz, the E group also showed significantly higher thresholds (E: 21.90 ± 19.66 dB; UE: 11.25 ± 13.94 dB; *p* = 0.002). All other frequencies evaluated in this ear showed no significant differences between groups (see Table 2). Right ear audiometric evaluation was performed using the same protocol (Figure 3B). On the other hand, there was no significant interaction between exposure and frequencies (two-way ANOVA; F (14, 732) = 0.541, *p* = 0.91). The results of Bonferroni’s multiple comparisons test are presented in Table 2. The SISI test analysis revealed no significant interaction between frequencies and pesticide exposure in either ear. In the left ear, exposure showed no significant effect on frequency responses (ANOVA, F (4, 245) = 0, *p* = 0.78). Bonferroni’s multiple comparisons test showed no significant differences between groups at 0.5 kHz (*p* = 0.46), 1 kHz (*p* > 0.99), 2 kHz (*p* > 0.99), 4 kHz (*p* > 0.99), and 8 kHz (*p* > 0.99). Similarly, in the right ear, exposure showed no significant effect on frequencies (F (4, 245) = 0, *p* = 0.83p), with Bonferroni’s multiple comparisons test showing no significant differences between groups at 0.5 kHz (*p* > 0.9999), 1 kHz (*p* > 0.9999), 2 kHz (*p* > 0.9999), 4 kHz (*p* > 0.9999), and 8 kHz (*p* = 0.3455).

### 3.2. Otoacoustic Emissions

Analysis of DPOAEs was conducted to assess cochlear function in E and UE groups. We quantified the total number of absent DPOAEs for each frequency in both ears, comparing the total of absent DPOEAs in each subject between E and UE groups. Shapiro–Wilk test for normality revealed non-normal distributions (E: *p* = 0.04, UE: *p* = 0.002). Analysis of DPOAE absence revealed differences between E and UE groups (Mann–Whitney U test, E: 2.50 ± 2.11, UE: 0.667 ± 0.985, *p* = 0.007, Figure 3B).

DPOAE amplitude analysis in the left ear showed no significant interaction between frequency and exposure (F (5, 274) = 0.30, *p* = 0.91). Bonferroni’s multiple comparisons test showed no significant differences between E and UE groups at any tested frequency: at 1 kHz (E: 11.42 ± 5.306 dB, n = 28; UE: 9.245 ± 4.912 dB, n = 20; *p* = 0.83), 1.5 kHz (E: 12.84 ± 4.747 dB, n = 30; UE: 11.36 ± 6.600 dB, n = 20; *p* > 0.99), 2 kHz (E: 9.984 ± 5.722 dB, n = 31; UE: 8.184 ± 3.893 dB, n = 19; *p* > 0.99), 3 kHz (E: 6.982 ± 4.513 dB, n = 28; UE: 6.370 ± 3.497 dB, n = 20; *p* > 0.99), 4 kHz (E: 8.862 ± 4.690 dB, n = 29; UE: 5.642 ± 4.158 dB, n = 19; *p* = 0.25), and 6 kHz (E: 5.246 ± 5.099 dB, n = 24; UE: 3.161 ± 4.315 dB, n = 18; *p* > 0.99). In the right ear, no significant interaction between frequency and exposure was found (F (5, 276) = 1.74, *p* = 0.1260). Bonferroni’s multiple comparisons test revealed no significant differences between E and UE groups at any frequency: at 1 kHz (E: 11.517 ± 5.306 dB, n = 30; UE: 12.985 ± 4.912 dB, n = 20; *p* > 0.9999), 1.5 kHz (E: 14.437 ± 4.747 dB, n = 30; UE: 11.095 ± 6.600 dB, n = 20; *p* = 0.1130), 2 kHz (E: 11.253 ± 5.722 dB, n = 30; UE: 10.295 ± 3.893 dB, n = 20; *p* > 0.9999), 3 kHz (E: 7.618 ± 4.513 dB, n = 28; UE: 6.350 ± 3.497 dB, n = 20; *p* > 0.9999), 4 kHz (E: 7.493 ± 4.690 dB, n = 29; UE: 8.305 ± 4.158 dB, n = 19; *p* > 0.9999), and 6 kHz (E: 3.344 ± 5.099 dB, n = 25; UE: 4.765 ± 4.315 dB, n = 17; *p* > 0.9999).

### 3.3. Impact of Pesticide Exposure on Auditory Brainstem Evoked Potentials (ABR)

**Figure 4:**
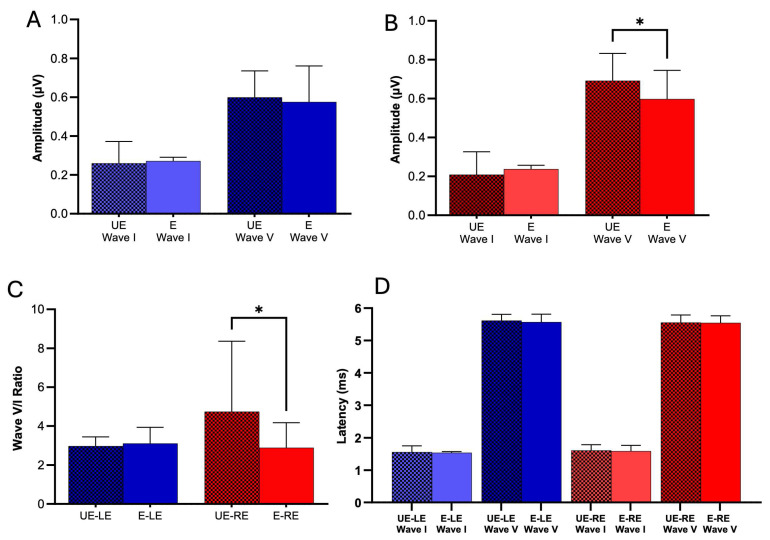
Auditory Brainstem Response (ABR) in E and UE Individuals. (**A**) Left ear peak-to-peak amplitudes. Peak-to-peak amplitudes of ABR waves I (left bars) and V (right bars) are shown for UE (UE, dotted) and E (E, solid) participants. Amplitude is measured in microvolts (μV). No statistically significant differences were observed between groups for either wave in the left ear. (**B**) Right ear peak-to-peak amplitudes. Same as (**A**), but for the right ear. Wave V amplitude in UE participants (black dotted bar) was significantly greater than in E participants (red dotted bar) (*p* < 0.05, *). (**C**) Wave V/I ratio: Ratio of wave V to wave I peak-to-peak amplitudes for left (LE) and right (RE) ears in both groups. The right ear V/I ratio was significantly higher in UE participants than in E participants (*p* < 0.05, *). No significant difference was observed for the left ear. (**D**) Right ear wave I and V latencies: Latencies (milliseconds) of waves I (solid bars) and V (dotted bars) in the right ear for both groups (UE, black; E, red). No statistically significant differences in latency were observed between groups for either wave. Error bars represent standard error of the mean (SEM). * *p* < 0.05. Abbreviations: ABR = auditory brainstem response; UE = unexposed; E = exposed; LE = left ear; RE = right ear.

The ABR technique allows us to assess the integrity of the auditory system at both the neural and subcortical levels. In this study, wave I (auditory nerve) and wave V (inferior colliculus) were analyzed to compare the values of latencies and peak amplitudes in both groups. We studied the peak-to-valley amplitude of wave I and wave V. Our results reveal that pesticide exposure associated with living near monoculture fields did not exert a significant influence on wave I and V peak-to-peak amplitude in the left ear (Figure 4A. ABR Wave I left ear: E: 0.272 ± 0.101, n = 28; UE: 0.259 ± 0.113, n = 17; Unpaired *t*-test, *p* = 0.70. ABR Wave V left ear: E: 0.576 ± 0.185, n = 29; UE: 0.599 ± 0.136, n = 18; Unpaired *t*-test, *p* = 0.64). In the right ear, we found no significant differences in wave I; however, for wave V, UE subjects had a significantly greater amplitude than E subjects (Figure 4B. ABR Wave I right ear: E: 0.238 ± 0.103, n = 28; UE: 0.209 ± 0.117, n = 18; Unpaired *t*-test, *p* = 0.38. ABR Wave V right ear: E: 0.597 ± 0.148, n = 28; UE: 0.692 ± 0.140, n = 19; Mann–Whitney test, *p* = 0.01). Additionally, when analyzing the wave V/I ratio in the right ear, UE individuals showed a significantly higher ratio than those exposed to pesticides. In contrast, no significant changes were observed in the left ear (Figure 4C. Ratio Wave V/I right ear: E: 2.89 ± 1.27, n = 28; UE: 4.74 ± 3.62, n = 18; Mann–Whitney test, *p* = 0.03. Ratio Wave V/I left ear: E: 3.11 ± 4.36, n = 28; UE: 2.98 ± 1.96, n = 17; Mann–Whitney test, *p* = 0.42).

In the latency analysis, our results revealed that pesticide exposure associated with living near monoculture fields did not significantly influence wave I and V latency in the left ear (ABR Wave I left ear: E: 1.54 ± 0.182, n = 28; UE: 1.56 ± 0.192, n = 17; Unpaired *t*-test, *p* = 0.73. ABR Wave V left ear: E: 5.57 ± 0.246, n = 29; UE: 5.61 ± 0.196, n = 18; Mann–Whitney test, *p* = 0.36. Similarly, in the right ear, we obtained comparable results (Figure 4D. ABR Wave I right ear: E: 1.59 ± 0.182, n = 28; UE: 1.61 ± 0.179, n = 18; Unpaired *t*-test, *p* = 0.68. ABR Wave V right ear: E: 5.55 ± 0.215, n = 28; UE: 5.55 ± 0.236, n = 19; Unpaired *t*-test, *p* = 0.92).

## 4. Discussion

Pesticide exposure is a significant public health concern in Chile due to the intensive application of these agrochemicals and a relative lack of consistent regulatory oversight. This concern is particularly pronounced in the region under study, given its substantial contribution to national pesticide sales.

Consequently, the study of pesticide use in this region is critical. Furthermore, pesticide use, in general, can have detrimental acute and chronic effects on human health, impacting various organ systems [32]. Prior research has also documented associations between pesticide exposure and auditory system dysfunction [6,33,34,35,36,37], further emphasizing the importance of investigating the potential health impacts of this intensive pesticide use. The present study revealed several auditory dysfunctions in residents living near agricultural fields. These include elevated high-frequency auditory thresholds (Figure 3A), a greater number of absent otoacoustic emissions (Figure 3C), and reduced wave V amplitudes of auditory brainstem evoked potentials (Figure 4B).

### 4.1. High-Frequency Thresholds, Speech Disturbance in Noise, and Pesticide Exposure

Our findings revealed significant differences in auditory thresholds at 14 and 16 kHz between pesticide E and UE individuals in the left ear (Figure 3A). These results are consistent with animal model studies (guinea pigs), indicating that these agents primarily target the OHCs of the cochlear base, potentially impairing hearing sensitivity [35]. Supporting this, Reischl et al. [36], using squirrel monkeys, reported significant decreases in hearing thresholds between 0.5 and 6 kHz following 40 days of parathion exposure. The mechanisms by which pesticides damage hair cells are thought to involve inducing edema of the stria vascularis and promoting the secretion of pro-inflammatory cytokines and reactive oxygen species in the spiral ligament, leading to an inflammatory response and reduced cochlear blood flow. These reactive oxygen species and pro-inflammatory cytokines may then be translocated to cochlear hair cells, activating both extrinsic and intrinsic apoptotic cascades, which in turn can induce mitochondrial dysfunction and further drive apoptosis [37]. In humans, studies have suggested that pesticide exposure may increase the risk of hearing loss, particularly at higher frequencies, and may also reduce otoacoustic emission amplitudes and efferent suppression by contralateral noise [38,39]. A prospective US study of 366 adults aged 20–69, using multivariate linear and logistic regression analyses, investigated the relationship between hearing loss and decreasing pure tone averages [30]. We hypothesize that the observed changes in high-frequency auditory thresholds associated with pesticide exposure may contribute to speech processing difficulties in noise. These subtle effects, which have not yet been studied and are not readily apparent in our paradigm, deserve special attention, especially considering the young age of the population studied. More research is needed to explore the possible long-term impact of these high-frequency disturbances on speech understanding in noisy environments.

### 4.2. Does Exposure to Pesticides Impair the Outer Hair Cell Functions?

DPOAEs provide a noninvasive method to assess cochlear function, particularly the integrity of PHCs [40]. Our analysis revealed a significantly higher number of absent DPOAEs in the E group than in the UE group. These findings suggest an impact on OHC function in pesticide-exposed individuals, manifesting as a higher number of DPOAE absences. This pattern resembles observations in workers exposed to industrial noise [41] and older individuals with moderate presbycusis [42].

Interestingly, we did not find a significant difference in DPOAE amplitude. The nature of pesticide-induced cochlear damage might explain the absence of significant differences in DPOAE amplitudes at most frequencies between E and UE subjects. As our high-frequency audiometry results indicate (14 and 16 kHz thresholds), pesticide damage may primarily affect the basal turn of the cochlea, which corresponds to higher frequencies than those typically assessed by standard DPOAE protocols. This unexpected result warrants further investigation and may indicate the complex effects of pesticide exposure on cochlear function, suggesting that pesticide exposure may affect the OHCs, potentially altering the cochlear amplifier.

### 4.3. Electrophysiological Auditory Response and Exposure to Pesticides

Our results also showed a decrease in the amplitude of ABR wave V in the right ear of exposed subjects (Figure 4B). This finding is consistent with previous studies that report differences in some ABR parameters between E and UE adult subjects. Alcarás et al. [38] found significant differences in the amplitude of ABR waves in adults exposed to pesticides, which aligns with our study, where we observed a significant reduction in wave V amplitude in the right ear. Similarly, studies on newborns exposed to pesticides, such as that by Sturza et al. [43], also report alterations in ABR parameters, highlighting the sensitivity of the developing auditory system to these toxic agents. Moreover, Dassanayake et al. [44] demonstrated that pesticide exposure can affect electrophysiological responses, such as event-related potentials, suggesting a broader impact on neuronal function beyond ABR. Mora et al. [45] also found that cortical potentials are affected in exposed individuals, indicating that pesticides can alter auditory function at multiple levels of the nervous system.

When comparing and analyzing the V/I ratio, we found that UE subjects had a significantly higher ratio in the right ear than E subjects (Figure 4C). This contrasts with the central gain model proposed by Schaette and McAlpine [46], where an increase in wave V amplitude is produced by auditory input deprivation. In our study, the alteration would not be caused by decreased sensory input but by functional damage in both the cochlea and the auditory nerve induced by pesticides. In the latency analysis, our results revealed that living near monoculture fields did not significantly influence the latencies of waves I and V in the left ear (Figure 4D). Similarly, we obtained comparable results in the right ear, suggesting that although the amplitude of neural responses is affected in the right ear, the speed of neural transmission remains unchanged.

These results highlight the asymmetrical effects suffered by populations living near agricultural fields. The significant reduction in wave V amplitude and the V/I ratio in the right ear contrasts with the tonal audiometry results, which showed damage in the left ear. This may reflect different vulnerabilities within the auditory system, where pesticides affect both peripheral structures in the left ear and central auditory pathways in the right ear. Previous studies have suggested that pesticides can induce oxidative stress and inflammatory responses in cochlea, contributing to the observed neuronal damage [39,47].

This observed lateralization of effects can be attributed to three distinct physiological mechanisms. Firstly, pesticide-induced damage may manifest differently across auditory structures within the same individual due to varying accumulation patterns [48,49]. The lipophilic nature of many pesticides [50] allows them to cross the blood–brain barrier and distribute unevenly throughout the auditory system, influenced by tissue composition and blood flow dynamics. Secondly, hemispheric specialization in auditory processing could contribute to this asymmetry, as the left and right auditory pathways exhibit functional differences in humans [51,52]. Specifically, the finding that high-frequency audiometry revealed greater impairment in the left ear aligns with previous research suggesting a stronger olivocochlear efferent reflex in the right ear [53,54]. This reflex, known for its protective role against cochlear damage, could provide differential protection in our young study population, explaining the observed interaural discrepancies. Thirdly, variations in metabolic activity and detoxification capacity between different neural structures could lead to some auditory regions being more vulnerable than others within the same subject [48,49]. This is consistent with previous research demonstrating that toxicants can selectively damage specific structures within the auditory system; for example, aminoglycosides primarily affect outer hair cells, while carboplatin preferentially damages inner hair cells [55,56]. This structural selectivity, combined with the complex pathways of the auditory system [51,52,57,58,59,60], suggests that pesticides could simultaneously affect peripheral structures in one ear and central auditory pathways connected to the other ear within the same individual. While our study design cannot definitively pinpoint the exact mechanism behind these asymmetries, these findings underscore the complex nature of pesticide-induced auditory dysfunction and emphasize the need for comprehensive assessments of various auditory structures in future investigations.

A key strength of this study lies in its focus on a young population residing nearby but not directly working in agricultural fields. While previous research has often examined the effects of pesticide exposure on farm workers, where the confounding influence of occupational noise exposure is a significant factor, our findings suggest that mere proximity to monoculture fields using pesticides may be sufficient to induce measurable auditory changes. This distinction is crucial. By isolating the impact of environmental pesticide exposure associated with living near monoculture fields independent of occupational noise, our study provides compelling evidence for the potential vulnerability of individuals living in proximity to agricultural areas. This study underscores the urgent need for developing and implementing public health policies to protect the auditory health of populations residing near monoculture agricultural operations or forestry activities where pesticides are employed. These policies should prioritize preventative measures and regular monitoring to safeguard the hearing of these at-risk communities and mitigate the long-term consequences of environmental pesticide exposure. In the same sense, it is crucial to conduct further research to explore the underlying mechanisms and determine whether these auditory changes are reversible or indicative of long-term neuronal damage.

### 4.4. Limitations

We acknowledge an important limitation in our study regarding exposure assessment. While we established pesticide exposure associated with living near monoculture fields based on residential proximity, we did not have the capacity to measure specific pesticide types or concentrations through biomarkers such as urinary metabolites. This limitation prevents us from establishing dose–response relationships or identifying which specific agrochemicals might be most associated with the observed auditory effects. Additionally, our relatively small sample size (51 participants total, with 31 exposed and 20 unexposed individuals) may limit statistical power, particularly for detecting subtle effects or performing more detailed subgroup analyses. Future studies should also consider individuals who work in non-agricultural occupations but whose workplaces are located within the 400-m exposure radius from monoculture fields, as these populations may experience different exposure patterns and durations that could affect auditory outcomes. Incorporating biomonitoring in these diverse exposure scenarios would significantly strengthen the evidence base regarding which pesticides pose the greatest risk to auditory health and at what exposure thresholds these effects begin to manifest.

## 5. Conclusions

In this work, we have reported associations between pesticide exposure through residential proximity to monoculture fields and auditory function alterations. These associations were mainly observed in high-frequency audiometry, ABR wave amplitude, and the wave V/I ratio. These results suggest a potential relationship between environmental pesticide exposure and auditory system changes. In this sense, our findings emphasize the need for enhanced monitoring and protective measures for populations residing near agricultural areas where pesticides are extensively used. Additionally, these results suggest the potential benefit of including high-frequency audiometric thresholds and ABR testing in audiological assessments of people living near monoculture fields to better detect subtle changes in auditory function.

## Figures and Tables

**Table 1:** Demographic description of exposed and unexposed groups.

Characteristic	Exposed (n = 31)	Unexposed (n = 20)	*p*-Value
Age, mean years (SD)	25.94 (6.45)	28.2 (5.34)	0.152 (a)
Sex, n (%)			0.107 (b)
Male	10 (32.26)	11 (55)	
Female	21 (67.74)	9 (45)	
Mother’s educational level, n (%)			0.127 (b)
None	0 (0)	1 (5)	
Elementary school	1 (3.23)	1 (5)	
High school	11 (35.48)	12 (60)	
College or more	19 (61.29)	6 (30)	
Economic perception, n (%)			0.502 (b)
High	13 (41.94)	11 (55)	
Medium	16 (51.61)	7 (35)	
Low	2 (6.45)	2 (10)	
Alcohol intake, n (%)	17 (54.84)	12 (60)	0.716 (b)
Smoking, n (%)	7 (22.58)	6 (30)	0.553 (b)
Distance in meters from home to any crop, mean (SD)	281.23 (371.19)	2872.89 (3367.88)	<0.0001 * (c)

Statistical analysis applied: (a) Mann-Whitney test, (b) χ2, (c) *t*-test. SD: Standard Deviation. * Significant at *p*-value < 0.05.

**Table 2:** Comparison of Audiometric Hearing Thresholds Between Exposed and Unexposed Groups (E group n = 31; UE group n= 20).

Frequency (kHz)	Left Ear Exposed	Left Ear Unexposed	Left Ear *p*-Value	Right Ear Exposed	Right Ear Unexposed	Right Ear *p*-Value
0.15	7.903 ± 4.614	8.500 ± 6.902	>0.99	8.387 ± 5.383	7.250 ± 4.993	>0.99
0.25	6.129 ± 6.546	8.000 ± 7.327	>0.99	7.419 ± 6.308	7.250 ± 8.347	>0.99
0.5	7.097 ± 6.554	6.750 ± 7.482	>0.99	7.419 ± 6.174	8.500 ± 6.802	>0.99
1	6.613 ± 4.161	6.500 ± 5.155	>0.99	7.258 ± 5.138	6.500 ± 5.405	>0.99
2	4.839 ± 6.890	5.250 ± 6.781	>0.99	5.000 ± 4.830	6.000 ± 7.182	>0.99
3	6.290 ± 5.472	6.500 ± 9.333	>0.99	7.419 ± 6.816	7.250 ± 7.518	>0.99
4	4.677 ± 7.631	7.500 ± 9.389	>0.99	5.000 ± 6.708	4.750 ± 7.691	>0.99
6	8.065 ± 9.633	7.000 ± 6.156	>0.99	4.516 ± 5.966	6.750 ± 6.340	>0.99
8	10.645 ± 7.931	9.250 ± 6.935	>0.99	10.323 ± 8.557	10.750 ± 5.684	>0.99
9	9.516 ± 9.518	8.250 ± 7.304	>0.99	11.774 ± 8.221	9.750 ± 6.781	>0.99
10	10.968 ± 9.951	7.500 ± 7.164	>0.99	10.161 ± 8.415	9.500 ± 8.095	>0.99
11.2	12.903 ± 11.956	8.250 ± 8.926	>0.99	9.548 ± 9.999	8.750 ± 8.091	>0.99
12.5	10.323 ± 13.162	4.250 ± 10.422	0.42	10.000 ± 12.845	8.750 ± 10.497	>0.99
14	16.613 ± 16.451	7.750 ± 11.059	0.02 *	15.000 ± 16.683	10.250 ± 11.410	0.97
16	21.897 ± 19.658	11.250 ± 13.943	0.002 **	16.607 ± 18.056	12.000 ± 12.397	>0.99

* *p* < 0.05, ** *p* < 0.01. Statistical significance.

## Data Availability

The data presented in this study (audiometric data, DPOAE measurements, ABR recordings, and demographic information) can be requested by contacting the corresponding author. All shared data will be appropriately de-identified to protect participant privacy.

## Supplementary Materials

The following supporting information can be downloaded at: https://www.mdpi.com/article/10.3390/toxics13050375/s1, Hearing health survey (Spanish and translated version).

## Abbreviations

The following abbreviations are used in this manuscript:

WHO	World Health Organization
OP	Organophosphates
IHCs	Inner Hair Cells
OHCs	Outer Hair Cells
ABRs	Auditory Brainstem Responses
DPOAEs	Distortion Product Otoacoustic Emissions
GIS	Geographic Information System
PTA	Pure-Tone Average
SISI	Short Increment Sensitivity Index
SPL	Sound Pressure Level
E	E (group)
UE	UE (group)
RE	Right Ear
LE	Left Ear
GDP	Gross Domestic Product
SEM	Standard Error of the Mean
dB	Decibel
kHz	Kilohertz
Hz	Hertz
μV	Microvolts

## References

[1] Chadha S., Kamenov K., Cieza A. (2021). The World Report on Hearing, 2021. Bull World Health Organ.

[2] Hesse G. (2016). Innenohrschwerhörigkeit: Teil I: Prävalenz, Diagnostik Und Ätiologie. Laryngorhinootologie.

[3] Ranjdoost F., Ghaffari M.-E., Azimi F., Mohammadi A., Fouladi-Fard R., Fiore M. (2023). Association between Air Pollution and Sudden Sensorineural Hearing Loss (SSHL): A Systematic Review and Meta-Analysis. Environ. Res..

[4] Castellanos M.J., Fuente A. (2016). The Adverse Effects of Heavy Metals with and without Noise Exposure on the Human Peripheral and Central Auditory System: A Literature Review. Int. J. Environ. Res. Public. Health.

[5] Terreros G., Cifuentes-Cabello C., D’Espessailles A., Munoz F. (2025). Impact of Pesticide Exposure on Auditory Health: Mechanisms, Efferent System Disruption, and Public Health Implications. Toxicology.

[6] Ames R.G., Steenland K., Jenkins B., Chrislip D., Russo J. (1995). Chronic Neurologic Sequelae to Cholinesterase Inhibition among Agricultural Pesticide Applicators. Arch. Environ. Health Int. J..

[7] Körbes D., da Silveira A.F., Hyppolito M.Â., Munaro G. (2010). Organophosphate-Related Ototoxicity: Description of the Vestibulocochlear System Ultrastructural Aspects of Guinea Pigs. Braz. J. Otorhinolaryngol..

[8] Moshammer H., Khan A.W., Wallner P., Poteser M., Kundi M., Hutter H.-P. (2019). Validity of Reported Indicators of Pesticide Exposure and Relevance for Cytotoxic and Genotoxic Effects on Buccal Cells. Mutagenesis.

[9] Hutter H.-P., Poteser M., Lemmerer K., Wallner P., Kundi M., Moshammer H., Weitensfelder L. (2021). Health Symptoms Related to Pesticide Use in Farmers and Laborers of Ecological and Conventional Banana Plantations in Ecuador. Int. J. Environ. Res. Public. Health.

[10] Moshammer H., Poteser M., Hutter H.-P. (2020). More Pesticides—Less Children?. Wien. Klin. Wochenschr..

[11] Petty C.S. (1958). Organic Phosphate Insecticide Poisoning. Am. J. Med..

[12] Crawford J.M., Hoppin J.A., Alavanja M.C.R., Blair A., Sandler D.P., Kamel F. (2008). Hearing Loss among Licensed Pesticide Applicators in the Agricultural Health Study. J. Occup. Environ. Med..

[13] Rabinowitz P.M., Sircar K.D., Tarabar S., Galusha D., Slade M.D. (2005). Hearing Loss in Migrant Agricultural Workers. J. Agromed..

[14] Dundar M.A., Derin S., Aricigil M., Eryilmaz M.A. (2016). Sudden Bilateral Hearing Loss after Organophosphate Inhalation. Turk. J. Emerg. Med..

[15] Guida H.L., Morini R.G., Cardoso A.C.V. (2010). Avaliação Audiológica Em Trabalhadores Expostos a Ruído e Praguicida. Braz. J. Otorhinolaryngol..

[16] Teixeira C.F., Brandão M.F.A. (1998). Efeitos Dos Agrotóxicos No Sistema Auditivo Dos Trabalhadores Rurais. Cad Inf Prev Acid.

[17] Perry M.J., May J.J. (2005). Noise and Chemical Induced Hearing Loss. J. Agromed..

[18] Prakash Krishnan Muthaiah V., Ding D., Salvi R., Roth J.A. (2017). Carbaryl-Induced Ototoxicity in Rat Postnatal Cochlear Organotypic Cultures. Environ. Toxicol..

[19] Lobarinas E., Salvi R., Ding D. (2016). Selective Inner Hair Cell Dysfunction in Chinchillas Impairs Hearing-in-Noise in the Absence of Outer Hair Cell Loss. JARO.

[20] Kujawa S.G., Liberman M.C. (2009). Adding Insult to Injury: Cochlear Nerve Degeneration after “Temporary” Noise-Induced Hearing Loss. J. Neurosci..

[21] Salvi R.J., Ding D., Wang J., Jiang H.-Y. (2000). A Review of the Effects of Selective Inner Hair Cell Lesions on Distortion Product Otoacoustic Emissions, Cochlear Function and Auditory Evoked Potentials. Noise Health.

[22] Helleman H.W., Dreschler W.A. (2012). Overall versus Individual Changes for Otoacoustic Emissions and Audiometry in a Noise-Exposed Cohort. Int. J. Audiol..

[23] Plack C.J., Barker D., Prendergast G. (2014). Perceptual Consequences of “Hidden” Hearing Loss. Trends Hear..

[24] Lobarinas E., Salvi R., Ding D. (2013). Insensitivity of the Audiogram to Carboplatin Induced Inner Hair Cell Loss in Chinchillas. Hear. Res..

[25] Maison S.F., Usubuchi H., Liberman M.C. (2013). Efferent Feedback Minimizes Cochlear Neuropathy from Moderate Noise Exposure. J. Neurosci..

[26] Yin P., Fritz J.B., Shamma S.A. (2014). Rapid Spectrotemporal Plasticity in Primary Auditory Cortex during Behavior. J. Neurosci..

[27] (2021). Liliana Yáñez Barrios Región Del Libertador Bernardo O’Higgins.

[28] Fenske R.A., Lu C., Barr D., Needham L. (2002). Children’s exposure to chlorpyrifos and parathion in an agricultural community in central Washington State. Environ. Health Perspect..

[29] Servicio Agrícola Ganadero (SAG) (2020). Declaración de Ventas de Plaguicidas de Uso Agrícola Año 2019.

[30] Gatto M.P., Fioretti M., Fabrizi G., Gherardi M., Strafella E., Santarelli L. (2014). Effects of Potential Neurotoxic Pesticides on Hearing Loss: A Review. Neurotoxicology.

[31] Long L., Tang X. (2022). Exploring the Association of Organochlorine Pesticides Exposure and Hearing Impairment in United States Adults. Sci. Rep..

[32] Teixeira C.F., Augusto L.G.d.S., Morata T.C. (2003). Saúde Auditiva de Trabalhadores Expostos a Ruído e Inseticidas. Rev. Saude Publica.

[33] Rizk H.G., Lee J.A., Liu Y.F., Endriukaitis L., Isaac J.L., Bullington W.M. (2020). Drug-Induced Ototoxicity: A Comprehensive Review and Reference Guide. Pharmacotherapy.

[34] Lobarinas E., Spankovich C., Prell C.G. (2017). Le Evidence of “Hidden Hearing Loss” Following Noise Exposures That Produce Robust TTS and ABR Wave-I Amplitude Reductions. Hear. Res..

[35] Shim H.J., An Y.-H., Kim D.H., Yoon J.E., Yoon J.H. (2017). Comparisons of Auditory Brainstem Response and Sound Level Tolerance in Tinnitus Ears and Non-Tinnitus Ears in Unilateral Tinnitus Patients with Normal Audiograms. PLoS ONE.

[36] Finkler A.D., da Silveira A.F., Munaro G., Zanrosso C.D. (2012). Otoproteção Em Cobaias Expostas a Agrotóxico e Ginkgo Biloba. Braz. J. Otorhinolaryngol..

[37] Reischl P., Van Gelder G.A., Karas G.G. (1975). Auditory Detection Behavior in Parathion-Treated Squirrel Monkeys (Saimiri Sciureus). Toxicol. Appl. Pharmacol..

[38] Wu Y., Zou H. (2022). Research Progress on Mitochondrial Dysfunction in Diabetic Retinopathy. Antioxidants.

[39] Alcarás P.A.d.S., Zeigelboim B.S., Corazza M.C.A., Lüders D., Marques J.M., Lacerda A.B.M. (2021). de Findings on the Central Auditory Functions of Endemic Disease Control Agents. Int. J. Environ. Res. Public. Health.

[40] Sena T.R.R., Dourado S.S.F., Lima L.V., Antoniolli Â.R. (2018). The Hearing of Rural Workers Exposed to Noise and Pesticides. Noise Health.

[41] Veuillet E., Collet L., Duclaux R. (1991). Effect of Contralateral Acoustic Stimulation on Active Cochlear Micromechanical Properties in Human Subjects: Dependence on Stimulus Variables. J. Neurophysiol..

[42] Korres G.S., Balatsouras D.G., Tzagaroulakis A., Kandiloros D., Ferekidou E., Korres S. (2009). Distortion Product Otoacoustic Emissions in an Industrial Setting. Noise Health.

[43] Belkhiria C., Vergara R.C., Martín S.S., Leiva A., Marcenaro B., Martinez M., Delgado C., Delano P.H. (2019). Cingulate Cortex Atrophy Is Associated with Hearing Loss in Presbycusis with Cochlear Amplifier Dysfunction. Front. Aging Neurosci..

[44] Sturza J., Silver M.K., Xu L., Li M., Mai X., Xia Y., Shao J., Lozoff B., Meeker J. (2016). Prenatal Exposure to Multiple Pesticides Is Associated with Auditory Brainstem Response at 9 months in a Cohort Study of Chinese Infants. Environ. Int..

[45] Dassanayake T., Gawarammana I.B., Weerasinghe V., Dissanayake P.S., Pragaash S., Dawson A., Senanayake N. (2009). Auditory Event-Related Potential Changes in Chronic Occupational Exposure to Organophosphate Pesticides. Clin. Neurophysiol..

[46] Mora A.M., Baker J.M., Hyland C., Rodríguez-Zamora M.G., Rojas-Valverde D., Winkler M.S., Staudacher P., Palzes V.A., Gutiérrez-Vargas R., Lindh C. (2022). Pesticide Exposure and Cortical Brain Activation among Farmworkers in Costa Rica. Neurotoxicology.

[47] Schaette R., McAlpine D. (2011). Tinnitus with a Normal Audiogram: Physiological Evidence for Hidden Hearing Loss and Computational Model. J. Neurosci..

[48] Wu Y. (2023). A Review on the Ethical Issues in Neurotechnology. Theor. Nat. Sci..

[49] Carey J.L., Dunn C., Gaspari R.J. (2013). Central Respiratory Failure during Acute Organophosphate Poisoning. Respir. Physiol. Neurobiol..

[50] McConnell R., Delgado-Téllez E., Cuadra R., Tórres E., Keifer M., Almendárez J., Miranda J., El-Fawal H.A.N., Wolff M., Simpson D. (1999). Organophosphate Neuropathy Due to Methamidophos: Biochemical and Neurophysiological Markers. Arch. Toxicol..

[51] Magomedov K.E., Zeynalov R.Z., Suleymanov S.I., Tataeva S.D., Magomedova V.S. (2022). Calculation of Lipophilicity of Organophosphate Pesticides Using Density Functional Theory. Membranes.

[52] Zatorre R.J., Belin P. (2001). Spectral and Temporal Processing in Human Auditory Cortex. Cereb. Cortex.

[53] Tervaniemi M., Hugdahl K. (2003). Lateralization of Auditory-Cortex Functions. Brain Res. Rev..

[54] Durante A.S., Carvallo R.M.M. (2002). Contralateral Suppression of Otoacoustic Emissions in Neonates: Supresión Contralateral de Las Emisiones Otoacüsticas En Recién Nacidos. Int. J. Audiol..

[55] Khalfa S., Collet L. (1996). Functional Asymmetry of Medial Olivocochlear System in Humans. Towards a Peripheral Auditory Lateralization. Neuroreport.

[56] Ding D., Jiang H., Salvi R.J. (2010). Mechanisms of Rapid Sensory Hair-Cell Death Following Co-Administration of Gentamicin and Ethacrynic Acid. Hear. Res..

[57] Wang J., Ding D., Salvi R.J. (2002). Functional Reorganization in Chinchilla Inferior Colliculus Associated with Chronic and Acute Cochlear Damage. Hear. Res..

[58] Robles L., Delano P.H. (2008). Efferent System.

[59] Terreros G., Delano P.H. (2015). Corticofugal Modulation of Peripheral Auditory Responses. Front. Syst. Neurosci..

[60] Pandya D.N., Seltzer B., Petrides M., Cipolloni P.B. (2015). Auditory System. Cerebral Cortex: Architecture, Connections, and the Dual Origin Concept.

