# Supplementary material for: Impact of Pesticide Exposure on High-Frequency Auditory Thresholds and Cochlear Function in Young People Residing near Agricultural Areas
Source: Toxics. 2025 May 6;13(5):375. doi: 10.3390/toxics13050375 (PMC12115959; doi:10.3390/toxics13050375)
Supplement: Supplementary file 1 [file toxics-13-00375-s001.zip › toxics-3497118-supplementary.pdf]

Universidad de O'Higgins

Questionnaire:

Form Code:

You are being invited to participate in an important study that seeks to evaluate and understand the mechanisms associated with hearing loss caused by pesticides in marked in the project:

"Chronic exposure to anticholinesterase pesticides effects on Hidden Hearing Loss and auditory processing development."

Next, you will be asked some questions that seek to obtain information regarding the place where you have mostly lived (GPS location), your hearing health history, your life history, and to quantify your levels of exposure to pesticides. To do this, we will use some instruments and variables through general questions (age, sex, socioeconomic level (family), year of study and place of residence) based on questions from the national survey on quality of life and health.
There are also a few others to assess medical history related to hearing. These questions are based on the questionnaire developed by Liberman, which asks about experiences of acoustic trauma, medication, medical history, noise exposure, as well as the use of hearing protection.

REMEMBER THAT THIS INFORMATION IS CONFIDENTIAL, WE WILL NOT KNOW THE IDENTITY OF THE PERSON WHO ANSWERS IT. WE ONLY USE CODES THAT ARE KNOWN ONLY TO THE PERSON WHO IS TAKING THE SURVEY.

Survey Code:

I have read the study information and the information on data confidentiality and agree that the data will be used as indicated in the study information and in the informed consent and assent.

☐ Yes

Participant information

1.- Sex

☐ Man
☐ Women
☐ Other

2.- Age: ……………………..

3.- Career or High School: ………………………………………………………………………

4. Course: ……………………………………………………………………...……………

5. Father's education level (One option only): None / Initial Basic/

☐ Primary Education Secondary/
☐ Secondary Education
☐ Higher Education (Non-University /
☐ University / Postgraduate University)

6. Mother's education level (One option only):

☐ None / Initial
☐ Basic/Primary Education
☐ Secondary/Middle Education
☐ Higher Education (Non-University / University / Postgraduate University)

7. In your opinion, during the last 12 months, what level of financial, economic, money or money stress have you felt? (One option only)?

☐ Little or nothing
☐ Moderate
☐ High or much
☐ Don't know/No answer

8. How would you rate your current financial situation and that of your family in general? Would you say it is… (One option only)

☐ Good
☐ Neither good nor bad
☐ Bad
☐ Don't know/No answer

Survey Code:

II. Medical history related to ear and hearing

1. **Have you ever used hearing aids in your life?**

☐ No ☐ Yes

2. **Have you experienced auditory trauma such as eardrum rupture?**

☐ No ☐ Yes ☐ Don't know

If your answer is Yes, indicate at what age ________________________________

Which ear was affected (circle): Left Right Both

3. **Have you ever done any work where you have been routinely exposed to noise, such as construction, artillery, military activities, or work in industry or with machines in the agricultural sector?**

☐ No ☐ Yes

If your answer is Yes, for how long?

____

If you have used hearing protection at work, what percentage of the time did you use hearing protection?

____

Describe your work ____

4. **Have you done any recreational activities that involve loud noises, such as hunting, handling machinery, carpentry or frequently attending concerts or discotheques?**

☐ No ☐ Yes

If your answer is Yes, for how long? ________________________________

Describe your recreational activity ____

5. Have you experienced temporary noises (ringing, buzzing, hissing, etc…) after exposure to loud noises?

☐ No ☐ Yes

If your answer is Yes, how long did you feel them? ________________________

Survey Code:

6. **Have you ever suffered from temporary hearing loss in your life?**

☐ No ☐ Yes

If your answer is Yes, for how long? _________________________________

If you know, tell us what caused that hearing loss.

____

7. **Do you feel like you hear in a distorted way?**

☐ No ☐ Yes

If your answer is Yes, explain to us what you mean.

____

8. **When you have a conversation in a noisy place, such as at home, restaurant, casino or classroom Can you understand what they are saying to you?**

☐ No ☐ Yes

If your answer is NO, explain to us why

____

9. **Have you suffered from sinusitis?**

☐ No ☐ Yes ☐ Don't know

10. **Have you suffered head injuries or a stroke?**

☐ No ☐ Yes ☐ Don't know

If your answer is Yes, Answer:

How old were you? __________________

What happened? __________________

Where were you diagnosed? __________________

Survey Code:

Indicate which symptom or symptoms accompanied the blow or trauma:

- [ ] Brief loss of consciousness
- [ ] Ringing in the ears
- [ ] Tinnitus
- [ ] Problems with memory or concentration
- [ ] Persistent headaches or weakness in the days following
- [ ] Dizziness or balance problems in the following days.

11. **Have you suffered accidents such as car crashes, falling from a horse or from a tractors?**

[ ] No [ ] Yes

If your answer is Yes, when did it happen?___________________________________

12. **Have you experienced earaches?**

[ ] No [ ] Yes

If your answer is Yes, on which side? : Right Left Both

If your answer is Yes, how long did it last? _____________________________

13. **Have you ever had blocked ears (for more than a few hours)?**

[ ] No [ ] Yes

If your answer is Yes, on which side? Right Left Both

If your answer is Yes, how long did it last? __________________________________

Survey Code:

14. **Have you been diagnosed with any of the following symptoms? (Check all that apply)**

- [ ] Generalized anxiety disorder
- [ ] Panic disorder
- [ ] Obsessive-compulsive disorder
- [ ] Post-traumatic stress
- [ ] Bipolar disorder or depression
- [ ] Schizophrenia
- [ ] Psychosis or hallucinations
- [ ] Meniere
- [ ] Fibromyalgia
- [ ] Multiple sclerosis
- [ ] Attention deficit
- [ ] Others you would like to indicate: ____
- [ ] Use of substances such as tobacco, alcohol, crack, marijuana.

Regarding substance use, tell us how many times a week you consume: ____________

Remember that all questions will be confidential and we will never know your identity.

15. **Have you ever experienced balance disorders in your life?**

☐ No ☐ Yes

If you answered Yes, please tell us how often you have experienced it:

- [ ] Sometimes
- [ ] Often
- [ ] Always

16. **Do you feel that some sounds irritate or bother you more than other people?**

☐ Always ☐ Many Times ☐ Almost Always ☐ Always

17. **Do you consider your hearing important for your daily activities?**

☐ No ☐ Yes

18. **Are you currently taking any medication?**

☐ No ☐ Yes

If your answer is Yes, what medicine is it? ___________________________________

Survey Code:

19. **During the last year, have you taken any medication for more than a month?**

☐ No ☐ Yes

If your answer is Yes, what medicine is it?___________________________________

20. **During the last year, have you taken any of the following medications permanently (for more than a week)?**

☐ Aspirin
☐ Indomethacin
☐ Naproxen
☐ Quinine
☐ Cisplatin
☐ Furesamide
☐ Bumetanide
☐ Streptomycin
☐ Gentomycin
☐ Neomycin
☐ OTHER

If your answer is Other, please indicate which medication was___________________

21. **Do you suffer or have you suffered as a child from frequent ear infections, such as Otitis?**

☐ No ☐ Yes

III. Farm work and pesticide exposure

1. **Have you ever worked as a farmer/field worker?** *ÿ If the answer is NO, skip to question* ***18***

☐ No ☐ Yes

2. How long have you worked as a farmer?

☐ 4 years or younger ☐ More than 4 years

3. Does anyone in your family work in agriculture?

☐ No ☐ Yes

Survey Code:

4. In which of the following positions do you work or have you worked in the last three years? years?

You must check all the options that fit your reality.

- [ ] Packaging of vegetables or fruits
- [ ] Land preparation, sowing or harvesting
- [ ] Selection, acquisition, preparation, disposal or transportation of pesticides
- [ ] Application of pesticides, pesticides or fertilizers
- [ ] Transport of fruits, vegetables or greens

5. Are you currently working in pesticide application?

- [ ] No
- [ ] Yes
- [ ] Not applicable

If yes, when was your last pesticide application?

- [ ] 2 years or more
- [ ] Less than 2 years
- [ ] Not applicable

6. Name the pesticides you apply at work:

- [ ] Chlorpyrifos (Lorsban, Troy)
- [ ] Methamidophos (MTD 600, Monitor, Tamaron)
- [ ] Metidazon
- [ ] Diazinon
- [ ] Fosmet
- [ ] Dimethoate
- [ ] Profenofos
- [ ] Cadusafos
- [ ] Other Organophosphates (Name them) ___________________________

7. Do you mainly work only in one season (seasonal) or are you an applicator? permanent pesticides?

- [ ] Seasonal
- [ ] Permanent
- [ ] Not applicable

8. How many years have you applied pesticides?

- [ ] 4 years or younger
- [ ] More than 4 years

Survey Code:

9. Do you eat, drink or smoke during application?

☐ No ☐ Always or occasionally ☐ Not applicable

10. During or after applying pesticides, do you wash your hands before smoking, eating or drinking?

☐ No ☐ Always or occasionally ☐ Not applicable

11. In addition to applying pesticides, do you mix and prepare them?

☐ No ☐ Yes ☐ Not applicable

12. The place where the pesticide is mixed is...

☐ Open ☐ Closed ☐ Not applicable

13. Do you use personal protective equipment when mixing?

☐ No ☐ Yes ☐ Not applicable

14. Do you change your clothes after application?

☐ No ☐ Yes or occasionally ☐ Not applicable

15. If you change clothes after work, where do you do it?

☐ Job ☐ Home ☐ Not applicable

16. How long does it take from the end of the application to taking a shower or bath?

☐ Less than 15 minutes ☐ 15 minutes or more ☐ Not applicable

17. When you work as a farmer, do you use the following equipment? personal protective equipment (PPE):

	Always	Never or occasionally
Use of PPE on hands (gloves)		
Use of PPE on the head (hat)		
Use of protective glasses (glasses, face shield or goggles) security)		
Use of respiratory PPE (respiratory mask with filters) recommended and facial protection)		
Use of PPE on the body (waterproof suit without cuts or holes)		
Use of PPE on feet (rubber boot)		

Survey Code:

18. Do you have a greenhouse, garden or field at home?

☐ No ☐ Yes

19. Approximate distance from the farms to your house (in meters)

☐ More than 500 meters (more than 5 blocks) ☐ 500 meters or less (less than 5 blocks)

20. Do you use insecticides, fungicides, herbicides, acaricides in your home?

☐ No ☐ Yes

If your answer is "yes", please tell us the name of the product: ______________________

IV. Lifestyles

REMEMBER THAT THIS INFORMATION IS CONFIDENTIAL, WE WILL NOT KNOW THE IDENTITY OF THE PERSON WHO ANSWERS IT. WE ONLY USE CODES THAT ARE KNOWN ONLY TO THE PERSON WHO IS TAKING THE SURVEY.

1. **Do you consume alcoholic beverages?**

☐ No ☐ Yes

How many drinks a week?:_______

2. **Do you smoke?**

☐ No ☐ Yes

How many cigarettes do you smoke a week?:_______

3. **In general, how do you consider your health to be?**

☐ Excellent
☐ Very good
☐ Good
☐ Regular
☐ Bad

V. Housing information

1. Current residence:

Street:………………………………………………………. ; Number: …………

Near which street?

Survey Code:

Commune: ……………………………………………………………….

City: …………………………………………………………

Region: ………………………………………………………….

2. Tell us where you lived 2 years ago

In the same house or apartment: ☐

If it is in another place, please provide us with the following information:

Street:………………………………………………………. ; Number: ………..

Near which street?

Commune: ……………………………………………………………….

City: …………………………………………………………

Region: ………………………………………………………….

3. Place of birth:

Commune: ……………………………………………………………….

Sector: …………………………………………………………………..

City: …………………………………………………………

Region: ………………………………………………………….

Usted está siendo invitado a participar en un importante estudio que busca evaluar y comprender los mecanismos asociados a la perdida auditiva generada por pesticidas en marcado en el proyecto:

" Chronic exposition to anticholinesterase pesticides' effects on Hidden Hearing Loss and auditory processing development."

A continuación, a usted se le realizarán algunas preguntas que buscan obtener información que permita obtener información respecto al lugar que usted ha habitado mayoritariamente (localización GPS), historial de su salud auditiva, historial de vida y la cuantificar sus niveles de exposición a pesticidas. Para ello, utilizaremos algunos instrumentos y variables a través de preguntas generales (edad, sexo, nivel socioeconómico (familia), año de estudio y lugar de residencia) basadas en preguntas de la encuesta nacional de calidad de vida y salud. También algunas otras para evaluar la historia médica relacionada con su audición. Estas preguntas se basan en el cuestionario elaborado por Liberman, que indaga sobre experiencias de traumas acústicos, medicación, historial médico, exposición a ruidos, así como uso de protección auditiva.

RECUERDA QUE ESTA INFORMACIÓN ES CONFIDENCIAL, NO CONOCEREMOS LA IDENTIDAD DE QUIEN LO RESPONDE. SOLO USAMOS CODIGOS QUE SON CONOCIDOS SOLO POR QUIÉN TE APLICA LA ENCUESTA.

Código Encuesta:

He leído la información del estudio y la información sobre la confidencialidad de los datos y acepto que los datos se utilizarán como se indica en la información del estudio y en el consentimiento y asentimiento informado.	**Si**

I. Información del participante

1.- Sexo

- [ ] Hombre
- [ ] Mujer
- [ ] Otro

2.- Edad: ……………………..

3.- Carrera o Liceo: ………………………………………………………………….

4. Curso: ……………………………………………………………...…………….

5. Nivel de escolaridad de su padre (Una sola opción):

- [ ] Ninguno / inicial
- [ ] Educación Básica/Primaria
- [ ] Educación Media /Secundaria
- [ ] Educación Superior (No Universitaria /Universitaria / Universitaria de Postgrado)

6. Nivel de escolaridad de su madre (Una sola opción):

- [ ] Ninguno / inicial
- [ ] Educación Básica/Primaria
- [ ] Educación Media /Secundaria
- [ ] Educación Superior (No Universitaria /Universitaria / Universitaria de Postgrado)

7. A su juicio durante los últimos 12 meses ¿Qué nivel de estrés financiero, económico, de dinero o plata ha sentido Ud.? (Una sola opción)?

- [ ] Poco o nada
- [ ] Moderado
- [ ] Alto o mucho
- [ ] No sabe/No responde

8. ¿Cómo calificaría en general su situación económica actual y la de su familia? Diría usted que es… (Una sola opción)

- [ ] Buena
- [ ] Ni buena ni mala
- [ ] Mala
- [ ] No sabe/No responde

Código Encuesta:

II. Historia médica relacionada con el oído y la audición

1. **¿Has utilizado audífonos alguna vez en tu vida?**

☐ No ☐ Si

2. **¿Has experimentado trauma auditivo tal como ruptura timpánica?**

☐ No ☐ Si ☐ No lo Sé

Si tu respuesta es Si, Indica a qué edad ________________________________

Cuál oído fue el afectado (encierre en un círculo): Izquierdo Derecho Ambos

3. **¿Has realizado trabajos donde rutinariamente hayas estado expuesto a ruidos, tal como construcción, artillería, actividades militares, o trabajo en industria o con máquinas en el sector de agricultura?**

☐ No ☐ Si

Si tu respuesta es Si, ¿Por cuánto tiempo?

____

Si has utilizado protección auditiva en tu trabajo, ¿Qué porcentaje de tiempo utilizaste protección auditiva? _____________

Describe tu trabajo ____

4. **¿Has realizado alguna actividad recreativa que involucre ruidos fuertes, tal como caza, manejo de maquinaria, carpintería o asistido frecuentemente a conciertos o Discotheque?**

☐ No ☐ Si

Si tu respuesta es Si, ¿Por cuánto tiempo? ________________________________

Describe tu actividad recreativa ____

5. **¿Has experimentado ruidos temporales (timbre, zumbidos, siseos, etc…) después de exposición a ruidos fuertes?**

☐ No ☐ Si

Si tu respuesta es Si, ¿Por cuánto tiempo los sentiste? _______________________

Código Encuesta:

6. **¿Alguna vez en tu vida has sufrido pérdida auditiva temporal?**

☐ No ☐ Si

Si tu respuesta es Si, ¿Por cuánto tiempo? ________________________________

Si lo sabes indícanos cuál fue la causa de esa pérdida auditiva

____

7. **¿Te parece que escuchas de forma distorsionada?**

☐ No ☐ Si

Si tu respuesta es Si, explícanos a qué refieres

____

8. **Cuando sostienes una conversación en un lugar ruidoso, tal como en el hogar, restaurant, casino o sala de clases ¿Puedes entender lo que te están diciendo?**

☐ No ☐ Si

Si tu respuesta es NO, explica nos a qué te refieres____

9. **¿Has sufrido sinusitis?**

☐ No ☐ Si ☐ No lo Se

10. **¿Has sufrido golpes en la cabeza o derrame cerebral?**

☐ No ☐ Si ☐ No lo Sé

Si tu respuesta es Si, Responda:

¿Qué edad tenías? _________________

¿Qué ocurrió? _________________

¿Dónde fuiste diagnosticado? _________________

Código Encuesta:

Indica qué síntoma o síntomas acompañó el golpe o trauma:

☐ Perdida breve de conciencia
☐ Zumbidos en los oídos
☐ Tinnitus
☐ Problemas en la memoria o concentración
☐ Dolores de cabeza o debilidad persistentes los días posteriores
☐ Mareos o problemas de equilibrio los días posteriores.

11. **¿Has sufrido accidentes tal como choques de automóviles, caída de caballo o de tractores?**

☐ No ☐ Si

Si tu respuesta es Si, ¿Cuándo ocurrió?___________________________________

12. **¿Has experimentado dolores de oído?**

☐ No ☐ Si

Si tu respuesta es Si, ¿En qué lado? : Derecho Izquierdo Ambos

Si tu respuesta es Si, ¿Cuánto duro? ___________________________

13. **¿Has sentido de forma permanente (por más de algunas horas) los oídos tapados?**

☐ No ☐ Si

Si tu respuesta es Si, ¿En qué lado? Derecho Izquierdo Ambos

Si tu respuesta es Si, ¿Cuánto duro? _______________________________

Código Encuesta:

14. **¿Has sido diagnosticado con alguno de los siguientes síntomas? (Marque todos los que correspondan)**

- [ ] Desorden de ansiedad generalizada
- [ ] Crisis de pánico
- [ ] Trastorno obsesivo compulsivo
- [ ] Estrés post-traumático
- [ ] Desorden bipolar o depresión
- [ ] Esquizofrenia
- [ ] Psicosis o alucinaciones
- [ ] Meniere
- [ ] Fibromialgia
- [ ] Esclerosis multiple
- [ ] Déficit Atencional
- [ ] Otros que nos quieras indicar: ____
- [ ] Uso de sustancias tales tabaco, alcohol, pasta base, marihuana.

Respecto al uso de sustancia indicanos cuantas veces a la semana consumes: ____________

Recuerda que Todas las preguntas serán confidenciales y no conoceremos nunca tu identidad.

15. **¿Alguna vez en la vida has experimentado desordenes del equilibrio?**

[ ] No   [ ] SI

Si has respondido Si, indícanos con qué frecuencia lo has experimentado:

- [ ] A veces
- [ ] A menudo
- [ ] Siempre

16. **¿Sientes que algunos sonidos te irritan o molestan más que a las demás personas?**

[ ] Siempre   [ ] Muchas Veces   [ ] Casi Siempre   [ ] Siempre

17. ¿**Tu audición la consideras importante para tus actividades diarias?**

[ ] No   [ ] Si

18. **¿Estas actualmente tomando alguna medicación?**

[ ] No   [ ] Si

Si tu respuesta es Si, ¿qué medicamento es? ___________________________________

Código Encuesta:

19. **Durante el último año ¿Has tomado por más de un mes alguna medicación?**

☐ No ☐ Si

Si tu respuesta es Si, ¿qué medicamento es?___________________________________

20. **Durante el último año ¿Has tomado de forma permanente (por más de una semana) alguno de los siguientes medicamentos?**

- ☐ Aspirina
- ☐ Indometacina
- ☐ Naproxeno
- ☐ Quinina
- ☐ Cisplatino
- ☐ Furesamida
- ☐ Bumetanida
- ☐ Estreptomicina
- ☐ Gentomicina
- ☐ Neomicina
- ☐ OTRO

Si tu respuesta es Otro, por favor indique, cuál medicamento fue___________________

21. **Sufres o has sufrido cuando niño(a) infecciones auditivas frecuentes, tales como Otitis**

☐ No ☐ Si

III. Trabajo agrícola y exposición a pesticidas

1. **¿Alguna vez ha trabajado como agricultor/a o en el campo?** → *Si la respuesta es NO, saltar a la pregunta* ***18***

☐ No ☐ Si

2. ¿Cuánto tiempo has trabajado como agricultor/a?

☐ 4 años o menos ☐ Más de 4 años

3. ¿Alguien de su familia trabaja en la agricultura?

☐ No ☐ Si

Código Encuesta:

4. **¿En cuál de las siguientes funciones trabaja o has trabajado en los últimos tres años?**

Debe marcar todas las opciones que se ajusten a su realidad.

- [ ] Empaque de hortalizas o frutas
- [ ] Preparación de la tierra, siembra o cosecha
- [ ] Elección, adquisición, preparación, desecho o transporte de plaguicidas
- [ ] Aplicación de pesticidas, plaguicidas o abonos
- [ ] Transporte de frutas, verduras o hortalizas

5. **¿Actualmente estás trabajando aplicando pesticidas?**

[ ] No [ ] Si [ ] No aplica

Si la respuesta es sí, ¿Cuando fue su última aplicación de pesticida?

[ ] 2 años o más [ ] Menos de 2 años [ ] No aplica

6. **Nombra los pesticidas que aplicas en el trabajo:**

- [ ] Clorpirifos (Lorsban, Troya)
- [ ] Methamidophos (MTD 600, Monitor, Tamaron)
- [ ] Metidazon
- [ ] Diazinon
- [ ] Fosmet
- [ ] Dimetoato
- [ ] Profenofos
- [ ] Cadusafos
- [ ] Otros Organofosforados (Nombrelos) ___________________________

7. **¿Trabajas principalmente solo en una temporada (estacional) o eres aplicador permanente de pesticidas?**

[ ] Estacional [ ] Permanente [ ] No aplica

8. **¿Cuántos años has aplicado pesticidas?**

[ ] 4 años o menos [ ] Más de 4 años

Código Encuesta:

9. ¿Comes, bebes o fumas durante la aplicación?

☐ No ☐ Siempre u ocasionalmente ☐ No aplica

10. Durante o después de la aplicación de pesticidas, ¿te lavas las manos antes de fumar, comer o beber?

☐ No ☐ Siempre u ocasionalmente ☐ No aplica

11. Además de aplicar pesticidas, ¿los mezcla y preparas?

☐ No ☐ Si ☐ No aplica

12. El lugar donde se mezcla el pesticida es ...

☐ Abierto ☐ Cerrado ☐ No aplica

13. ¿Usted usa equipo de protección personal cuando mezcla?

☐ No ☐ Si ☐ No aplica

14. ¿Te cambias de ropa después de la aplicación?

☐ No ☐ Si u ocasionalmente ☐ No aplica

15. Si te cambias de ropa después del trabajo, ¿dónde lo haces?

☐ Trabajo ☐ Hogar ☐ No aplica

16. ¿Cuánto tiempo lleva entre el final de la aplicación y tomar una ducha o baño?

☐ Menos de 15 minutos ☐ 15 minutos o más ☐ No aplica

17. Cuando realizas trabajo como agricultor/a, ¿utilizas el (los) siguiente (s) equipo (s) de protección personal (EPP):

	**Siempre**	**Nunca u ocasional mente**
Uso de EPP en manos (guantes)		
Uso de PPE en la cabeza (sombrero)		
Uso de gafas protectoras (gafas, careta o gafas de seguridad)		
Uso de EPP respiratorio (máscara respiratoria con filtros recomendados y protección facial)		
Uso de PPE en el cuerpo (traje impermeable sin cortes ni agujeros)		
Uso de PPE en los pies (bota de goma)		

Código Encuesta:

18. ¿Tienes un invernadero, huerto o campo en casa?

☐ No ☐ Si

19. Distancia aproximada de las granjas a su casa (en metros)

☐ Más de 500 metros (más de 5 cuadras) ☐ 500 metros o menos (menos de 5 cuadras)

20. ¿Usan insecticidas, fungicidas, herbicidas, acaricidas en tú casa?

☐ No ☐ Si

Si tu respuesta es "si", indícanos el nombre del producto: ______________________

IV. Estilos de vida

RECUERDA QUE ESTA INFORMACIÓN ES CONFIDENCIAL, NO CONOCEREMOS LA IDENTIDAD DE QUIEN LO RESPONDE. SOLO USAMOS CODIGOS QUE SON CONOCIDOS SOLO POR QUIÉN TE APLICA LA ENCUESTA.

1. **¿Consumes bebidas alcohólicas?**

☐ No ☐ Si

¿Cuantas bebidas a la semana?:_______

2. **¿Fumas?**

☐ No ☐ Si

¿Cuantos cigarrillos consumes a la semana?:_______

3. **En general, ¿Cómo consideras que es tu salud?**

☐ Excelente
☐ Muy buena
☐ Buena
☐ Regular
☐ Mala

V. Información de la vivienda

1. Residencia actual:

Calle:……………………………………………………… ; Número: …………

¿Cerca de qué calle?..

Código Encuesta:

Comuna: …………………………………………………………

Ciudad: …………………………………………………………

Región: …………………………………………………………

2. **Cuéntanos dónde vivías hace 2 años atrás**

En la misma casa o departamento: ☐

Si es en otro, indícanos los siguientes datos:

Calle:……………………………………………………… ; Número: …………

¿Cerca de qué calle?...

Comuna: …………………………………………………………

Ciudad: …………………………………………………………

Región: …………………………………………………………

3. **Lugar de nacimiento:**

Comuna: …………………………………………………………

Sector: …………………………………………………………..

Ciudad: …………………………………………………………

Región: …………………………………………………………
